# Chelerythrine potentiates meropenem activity against NDM-producing carbapenem-resistant Enterobacteriaceae

**DOI:** 10.3389/fvets.2026.1878928

**Published:** 2026-07-08

**Authors:** Tengfei Li, Yanhu Huang, Wei Liu, Genxi Zhang, Zhiqiang Wang, Xia Xiao

**Affiliations:** 1 Jiangsu Co-Innovation Center for Prevention and Control of Important Animal Infectious Diseases and Zoonoses, College of Veterinary Medicine, Yangzhou University, Yangzhou, China; 2 College of Animal Science and Technology, Yangzhou University, Yangzhou, China; 3 Institute of Comparative Medicine, Yangzhou University, Yangzhou, China; 4 Joint International Research Laboratory of Agriculture and Agri-Product Safety of Ministry of Education of China, Yangzhou University, Yangzhou, China

**Keywords:** helerythrine, meropenem, NDM, metallo-β-lactamase, antibiotic adjuvant

## Abstract

**Introduction:**

NDM-driven carbapenem-resistant Enterobacteriaceae (CRE) are spreading globally, posing a serious One Health threat that extends to veterinary medicine through transmission among food-producing animals, companion animals, and the farm-to-food continuum. Antibiotic adjuvants that restore or potentiate the activity of existing antibiotics represent a promising strategy to address this challenge.

**Methods:**

In this study, we evaluated the ability of the natural benzophenanthridine alkaloid chelerythrine to potentiate meropenem activity against NDM-producing Enterobacteriaceae. Antibacterial synergy was assessed using checkerboard assays, resistance development assays, Galleria mellonella infection models, and a murine thigh infection model. The underlying mechanism was investigated through NDM enzymatic activity assays, Zn^2+^ competition experiments, molecular docking, molecular dynamics simulations, and bacterial physiological assays measuring proton motive force, ATP production, and reactive oxygen species (ROS) generation.

**Results:**

Chelerythrine strongly potentiated meropenem activity against blaNDM-1-, blaNDM-5-, and blaNDM-9-positive Enterobacteriaceae, with FICI values ranging from 0.18 to 0.31. Chelerythrine markedly delayed the emergence of meropenem resistance. Combination therapy increased survival to 75% in infected Galleria mellonella larvae and significantly reduced bacterial burden in the murine thigh infection model. Mechanistically, chelerythrine inhibited NDM enzymatic activity by occupying the catalytic pocket and interacting with residues surrounding the dinuclear Zn^2+^ center. In addition, chelerythrine caused extensive bacterial cellular damage by destabilizing the proton motive force, reducing ATP levels, and promoting ROS accumulation.

**Discussion:**

These findings demonstrate that chelerythrine restores meropenem efficacy against NDM-producing Enterobacteriaceae through a dual mechanism involving suppression of metallo-*β*-lactamase activity and induction of metabolic disruption. Chelerythrine therefore represents a promising meropenem adjuvant for combating NDM-mediated carbapenem resistance.

## 1 Introduction

Antibiotics provide important protection against bacterial infections in intensive livestock and poultry production. However, the widespread use of antibiotics also causes antimicrobial resistance (AMR), which greatly impaired the therapeutic effectiveness of antibiotics. A major challenge to global public health is the rise and spread of multidrug-resistant (MDR) infections. Carbapenem-resistant Enterobacteriaceae (CRE) infections, which render many antibiotics, including carbapenems, ineffective (1), are a major public health concern (2–4). The production of carbapenemases, including class A serine carbapenemases, class B metallo-*β*-lactamases, and class D OXA-48–type serine carbapenemases, is a primary mechanism underlying carbapenemase resistance. Among these, blaNDM–encoded class B metallo-*β*-lactamases are among the most prevalent determinants in Enterobacteriaceae isolated from food and wildlife (5–8). There is an urgent need to develop new strategies to treat infections caused by blaNDM-positive Enterobacteriaceae.

Discovering new antimicrobial chemotherapies is an effective strategy for tackling antibiotic resistance. However, high development costs, long timelines, and low returns on investment have hindered the pace of antibacterial drug discovery. Since the late 1990s, the WHO has implemented several“antibiotic stewardship”policies within the One Health framework to extend antibiotic effectiveness (9, 10). Developing synergistic adjuvants is one such stewardship strategy to restore and enhance the efficacy of existing antibiotics (11, 12).

Plant metabolites are important sources of novel antibiotic adjuvants and antibacterial agents. For example, fisetin, a flavonoid commonly found in fruits and vegetables, restored the efficacy of meropenem against NDM-1–producing bacteria by inhibiting NDM-1 activity via binding to active-site residues Val73, Met248, and His250 (13). Likewise, corosolic acid, a pentacyclic triterpenoid derived from loquat leaves, kiwi roots, and hawthorn fruit, exhibited an inhibitory effect against the class A *β*-lactamase KPC-2, enhancing the bactericidal efficacy of imipenem and meropenem against MDR *E. coli* (14). These results highlight the potential of plant-derived compounds as antibiotic adjuvants and offer new approaches to combat bacterial resistance.

Chelerythrine, a natural benzophenanthridine alkaloid extracted from *Chelidonium majus* and *Macleaya cordata*, has been reported to exhibit insecticidal, analgesic, antibacterial, anti-inflammatory, and anticancer effects (15–17). Chelerythrine showed excellent antibacterial activity against Gram-positive bacteria, including methicillin-resistant *S.aureus* (MRSA), and extended-spectrum *β*-lactamase-producing *S.aureus* (ESBLs-SA), by disrupting DNA/protein synthesis and membrane permeability (18). Chelerythrine also restored colistin effectiveness against *mcr-1*–bearing pathogens by altering membrane fluidity, compromising respiratory function, and downregulating *mcr-1* expression (19). However, whether chelerythrine can synergize with carbapenems, particularly meropenem, against NDM-producing pathogens remains to be determined.

In this study, carbapenem-resistant Enterobacteriaceae were used to investigate the potential synergistic effects of chelerythrine and meropenem. The adjuvant mechanisms of chelerythrine were further explored by evaluating NDM-1 enzymatic activity, bacterial energy homeostasis, and oxidative stress. Finally, the safety and *in vivo* efficacy of chelerythrine as a carbapenem adjuvant were evaluated. These findings indicate that chelerythrine is a promising carbapenem antibiotic adjuvant and provide a viable strategy for treating infections caused by carbapenem-resistant Enterobacteriaceae in veterinary settings.

## 2 Experimental section

### 2.1 Chemicals and strains

Meropenem (Catalog no. B28410) and chelerythrine (Catalog no. S26109) were acquired from Shanghai Yuanye Bio-Technology Co., Ltd. (Shanghai, China). For subsequent usage, dissolve meropenem in water and chelerythrine in dimethyl sulfoxide, then stored at −20 °C. The following strains were maintained in this lab: *E. coli* B2 (*bla_NDM-5_*), *E. coli* HH194 (*bla_NDM-9_*), *E. coli* M593 (*bla_NDM-1_*), *E. coli* DH5α (*bla_KPC-2_*), *E. coli* DH5α (*bla_IMP-4_*), *K. oxytoca*BT6-1 (*bla_OXA-181_*), *P. aeruginosa*PA22-40 (*bla_IMP-10_*), *E.coli*DH5α (*bla_VIM-1_*), *K.pneumoniae*LR1-1-1 (*bla_NDM-5_*), *K.pneumoniae*LR11-2 (*bla_NDM-1_*), *E. coli* DH5α, *E. coli* K582, and ATCC 25922.

### 2.2 Checkerboard assays

The American Society for Clinical and Laboratory Standards’ recommendations for broth microdilution were followed in order to calculate the minimum inhibitory concentrations (MICs) of meropenem and chelerythrine for every strain. To evaluate the synergistic action of chelerythrine and meropenem, checkerboard tests were employed. The procedure involved filling each well of a 96-well plate with an 8 × 8 matrix with 100 μL of MHB. Next, two dilutions of meropenem and chelerythrine were made along the ordinate and abscissa, respectively. The absorbance of each well at 600 nm was measured following an 18-h incubation period at 37 °C with a bacterial suspension (1.5 × 10^6^ CFUs/mL). For the FIC index (FICI), the biological replicates’ mean absorbance at 600 nm was displayed. FIC index = FICI_a_ + FICI_b_ = MIC_ab_/MIC_a_ + MIC_ba_/MIC_b_. The FICI of ≤0.5 suggested synergy.

### 2.3 Time-dependent killing curves

LB was diluted with bacteria at a ratio of 1:1,000. The bacteria were then treated with either meropenem (16 μg/mL) and chelerythrine (64 μg/mL) alone or in combination, whereas the control groups received no medication treatment. At each time point (0, 4, 8, and 12 h), samples were serially diluted ten times and plated on LB plates. After the bacterial colonies were incubated overnight, the number of primary CFUs/mL was determined.

### 2.4 Resistance development study

Overnight cultures of *E. coli* B2 were diluted 1:100 in LB broth with either 1/4 × MIC of chelerythrine or 1/8 × MIC of meropenem. After 12 h of incubation at 37 °C, the bacterial culture was diluted 1:100 and moved to a new medicated media to begin the next generation. Every five passages, MIC values were calculated. For 16 days, the serial passage was carried out.

### 2.5 Enzyme kinetics

Protein purification was performed using the method described previously (20). In a 96-well flat-bottom plate, chelerythrine and NDM-1 enzyme diluent were co-incubated for 15 to 30 min at 37 °C. After adding nitrocefin, the mixture was incubated for 15 min at 37 °C. A microplate reader was used to quantify absorbance at 492 nm, with three biological replicates per group. Zn^2+^ was then added, and the experimental procedures were repeated.

### 2.6 Molecular docking and molecular dynamics simulation

Molecular docking of chelerythrine with NDM-1 was performed using AutoDock Vina. The NDM-1 structure was prepared by removing non-essential water molecules while retaining the catalytic Zn^2+^ ions, followed by hydrogen addition and charge assignment. The CHE structure was energy-minimized before docking. The docking grid was centered on the dinuclear Zn^2+^ catalytic pocket, and binding poses were analyzed based on docking score and interactions with active-site residues. For molecular dynamics simulation, the selected NDM-1–CHE complex was solvated, neutralized, energy-minimized, and equilibrated under NVT and NPT conditions. Production simulation was then performed, and the trajectory was analyzed by RMSD, RMSF, radius of gyration, SASA, and hydrogen-bond interactions to evaluate complex stability.

### 2.7 Measurement of PMF and ROS levels

Bacteria were labeled with BCECF-AM, DiSC3 (5), or DCFH-DA fluorescent probes to assess intracellular pH, membrane potential, or ROS levels in bacterial cells post-chelerythrine treatment, respectively. *E. coli* B2 cells were cultured, expanded, and resuspended prior to incubation with fluorescent probes at 37 °C for 30 min in darkness. Subsequently, varying concentrations of chelerythrine were introduced, followed by an additional incubation at 37 °C for 1 h under light-protected conditions. Fluorescence intensity was quantified using a microplate reader with designated excitation/emission wavelength pairs: 488 nm/535 nm (BCECF-AM), 622 nm/670 nm (DiSC3 (5)), and 488 nm/525 nm (DCFH-DA). Each experimental group included three biological replicates.

### 2.8 Measurement of NAD^+^/NADH and ATP levels

After an overnight growth period, *E. coli* B2 cultures were centrifuged, resuspended, and aliquoted into 2 mL centrifuge tubes. Cells were then exposed to different chelerythrine concentrations for 4 hours. Following treatment, samples underwent three rounds of PBS washing and resuspension before being centrifuged for five minutes at 4 °C and 5,000 × *g*. After discarding the supernatant, pellets were carefully pipetted back into a cold lysis buffer. After incubation on ice, centrifuge the lysate. For further examination, the resultant supernatant was gathered. Commercial NAD+/NADH assay kits and ATP detection kits were used to measure the amounts of NAD+/NADH and ATP, respectively.

### 2.9 Measurement of erythrocyte hemolysis rate

In a 96-well plate, the test medication was co-incubated with a 4% erythrocyte suspension. Following an hour of incubation at 37 °C, the plate was centrifuged for five minutes. The absorbance at 540 nm was then measured after 100 μL of the supernatant from each well was moved to a fresh 96-well plate. Calculation formula: Hemolysis rate (%) = (A-A0)/(Atotal-A0) × 100%.

### 2.10 Larval infection model

A single colony of *E. coli* B2 (*bla_NDM-5_*) was cultured overnight at 37 °C and 200 rpm. The culture was diluted 1:1,000, incubated for 4 h, and resuspended in PBS to an OD_600_ of 0.2. Galleria mellonella larvae (~300 mg) were acclimated at 37 °C for 4 h and randomly divided into four groups (*n* = 10). Larvae were infected by injecting 10 μL of bacterial suspension into the right posterior proleg. At 1 h post-infection, larvae received 10 μL of PBS, meropenem (MER, 0.7 mg/kg), chelerythrine (CHE, 4 mg/kg), or MER plus CHE via the left posterior proleg. Larvae were incubated at 37 °C for 2 days, and survival was recorded.

### 2.11 Bacterial infections

To test the bacterial burden on the muscles of the thighs, mice were given an intramuscular injection of *E. coli* B2 at a dose of 2 × 10^8^ CFUs. Every 12 h, mice received subcutaneous injections of PBS, chelerythrine (4 mg/kg), meropenem (0.7 mg/kg), or a combination of chelerythrine and meropenem. The thigh muscles were removed, homogenized, diluted with PBS, and plated on the LB plate for CFU enumeration at 37 °C after the mice were put to sleep 36 h after infection.

### 2.12 Statistics and reproducibility

All data was analyzed using GraphPad Prism version 9.0 and presented as mean ± SD. After the *in vitro* testing, a one-way or two-way ANOVA for multiple comparisons was performed. The calculated *p* < 0.05 was defined as a significant difference.

## 3 Results

### 3.1 Chelerythrine restores meropenem efficacy against blaNDM-carrying bacteria

**Figure 1:**
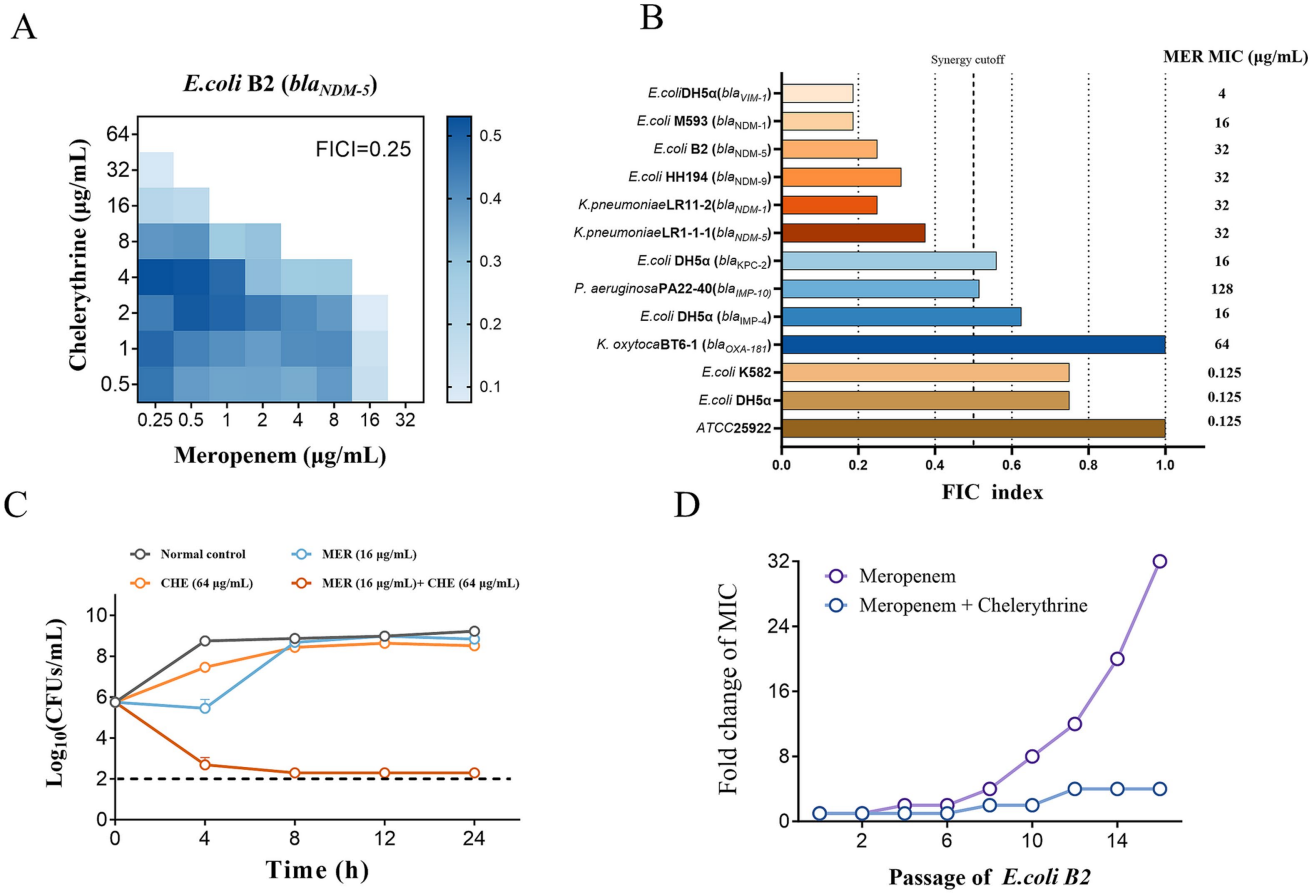
Synergistic antibacterial activity of chelerythrine in combination with meropenem. **(A)** Checkerboard broth microdilution assay showing the interaction between chelerythrine (CHE) and meropenem (MER) against *E. coli* B2 carrying blaNDM-5. **(B)** FICI values of the CHE–MER combination against meropenem-susceptible and carbapenem-resistant bacterial strains. Synergy was defined as FICI ≤ 0.5. The corresponding MER MIC values are indicated on the right. **(C)** Time-kill analysis of *E. coli* B2 exposed to CHE alone, MER alone, or the CHE–MER combination. **(D)** Changes in MER MIC during serial passage of *E. coli* B2 in the presence of MER alone or the CHE–MER combination, presented as fold change relative to the initial MIC. Data are representative of three independent experiments and shown as mean ± SD.

To assess the antibacterial activity of chelerythrine, minimum inhibitory concentrations (MICs) were determined using the broth microdilution method following the Clinical and Laboratory Standards Institute (CLSI) guideline M100. The results showed that the MICs of chelerythrine against all tested *E. coli* isolates ranged from 64 to 128 μg/mL (Supplementary Table 1), indicating weak antimicrobial activity against Gram-negative bacteria. However, pronounced synergy was observed between chelerythrine and meropenem against blaNDM-5–positive *E. coli* B2, with an FICI of 0.25 (Figure 1A). Similar synergistic effects were also observed in other *bla*_NDM_–carrying isolates, with FICI values of 0.18 for *E. coli* M593 (blaNDM-1–positive) and 0.31 for *E. coli* HH194 (blaNDM-9–positive) (Figure 1B). The combination was also effective against NDM-producing *Klebsiella pneumoniae* strains (Supplementary Figure S1). However, no synergistic effect was detected between chelerythrine and meropenem in meropenem-sensitive bacteria with the FICI value greater than 0.75. A weak synergistic effect was observed against meropenem-resistant isolates harboring other carbapenemase genes (e.g., KPC-2 or IMP), with FICI values ranging from 0.5 to 0.6. These results indicate that chelerythrine selectively restores meropenem susceptibility in NDM–producing bacteria (Figure 1B).

Figure 1C illustrates that monotherapy with either chelerythrine or meropenem displayed limited bactericidal activity, whereas their combination produced an approximately 6 log_10_ reduction in bacterial counts. These results further validated the robust synergy between chelerythrine and meropenem against NDM–producing bacteria. A serial-passage experiment was performed to determine whether chelerythrine affects the rate at which meropenem resistance emerges. The results showed that exposure to a sub-inhibitory concentration of meropenem led to a progressive increase in resistance, culminating in a 32-fold increase in MIC by the 16th passage. Conversely, the MIC in the combination treatment group increased by only 4-fold (Figure 1D), suggesting that co-treatment with chelerythrine markedly postponed the development of meropenem resistance.

### 3.2 Chelerythrine inhibits NDM by perturbing the dinuclear Zn active center

**Figure 2:**
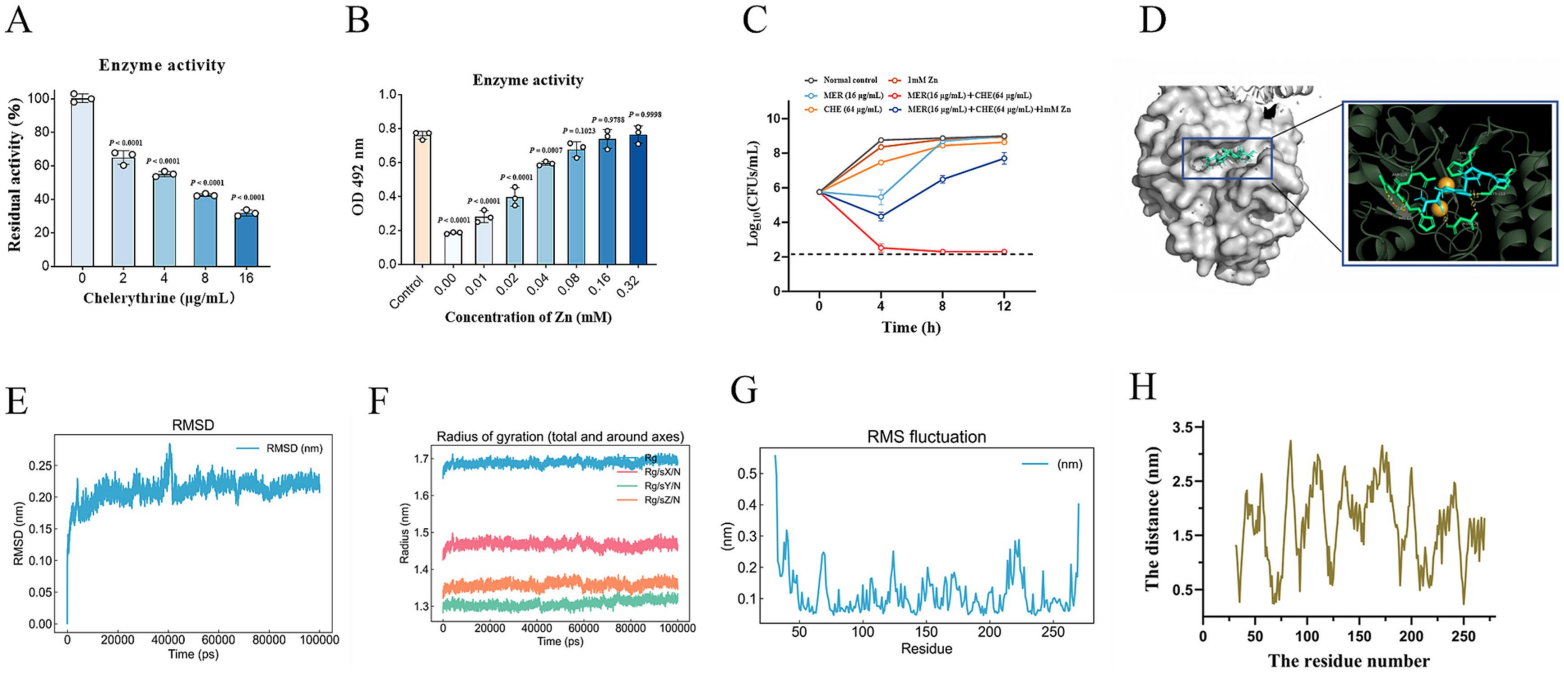
Chelerythrine inhibits NDM-1 activity and stably binds within the catalytic pocket. **(A)** Dose-dependent inhibition of NDM-1 hydrolytic activity by chelerythrine (CHE), determined using the nitrocefin hydrolysis assay. **(B)** Recovery of NDM-1 activity after supplementation with increasing concentrations of Zn^2+^. **(C)** Time–kill analysis showing that exogenous Zn^2+^ attenuates the bactericidal activity of the meropenem (MER)–CHE combination against NDM-1-producing bacteria. **(D)** Representative docking pose of CHE within the NDM-1 active site. **(E–G)** Molecular dynamics analysis of the NDM-1–CHE complex, including Cα root-mean-square deviation (RMSD) **(E)**, radius of gyration (Rg) **(F)**, and per-residue root-mean-square fluctuation (RMSF) **(G)**. **(H)** Time-dependent distances between CHE and key catalytic-site residues during the MD simulation. Data are presented as mean ± SD from three independent biological replicates. Statistical significance was determined by one-way ANOVA followed by Dunnett’s multiple-comparison test versus the untreated control; exact *p* values are shown in the figure.

Given that chelerythrine reinstated meropenem susceptibility in NDM–producing bacteria but not in meropenem-susceptible strains, we hypothesized that its synergistic activity may result from inhibition of NDM-1 (21, 22). Thus, the effect of chelerythrine (0–16 μg/mL) on NDM-1 enzymatic activity was assessed. A dose–dependent reduction in hydrolytic activity was observed in the presence of chelerythrine (Figure 2A), indicating that chelerythrine inhibits NDM-1 activity.

The functionality of NDM-1 relies on two Zn^2+^ ions at its catalytic site (23, 24). To determine whether chelerythrine inhibits NDM-1 by interfering with Zn^2+^, we evaluated its inhibitory effect on NDM-1 in the presence of supplemented Zn^2+^. The results demonstrated that Zn^2+^ addition alleviated chelerythrine-mediated inhibition in a dose–dependent manner (Figure 2B), indicating that chelerythrine inhibited NDM-1 through Zn^2+^–dependent interference at the catalytic site. Zn^2+^ supplementation markedly diminished the synergistic activity of chelerythrine and meropenem, as evidenced by approximately 7 log_10_ higher CFU/mL after 12 h of treatment (Figure 2C).

Molecular docking results showed that chelerythrine occupied the catalytic pocket of NDM-1, with the most favorable binding pose positioned at the dinuclear zinc center (Figure 2D). In this pose, chelerythrine formed metal-proximal contacts with Zn302 and Zn303 and was further stabilized by surrounding active-site residues, including His122, His189, Lys211, Gly219, Asn220, and His250, together with hydrophobic contacts involving Val73 and Trp93. The MD simulation further supported the stability of the chelerythrine–NDM-1 complex. Docking analyses of chelerythrine with NDM-5 and NDM-9 also supported this conclusion (Supplementary Figure S2). After an initial equilibration phase, the backbone RMSD reached a stable plateau at approximately 0.20–0.23 nm and remained within this range for most of the trajectory, with only a transient fluctuation around 40 ns (Figure 2E). The radius of gyration remained essentially constant throughout the simulation, indicating that chelerythrine binding did not induce major global conformational changes in NDM-1 (Figure 2F). RMSF analysis showed that residue mobility was generally low, with relatively higher fluctuations confined mainly to the terminal regions and several solvent-exposed loops, whereas the active-site region remained comparatively rigid (Figure 2G). Residue–ligand distance analysis revealed that chelerythrine maintained preferential contacts with specific catalytic pocket residues during the simulation, primarily Asp124, Lys211, and His250 (Figure 2H). These results indicate that chelerythrine forms a stable complex with NDM-1 by occupying the catalytic pocket and engaging residues surrounding the dinuclear Zn center.

### 3.3 Chelerythrine disrupts bacterial energy homeostasis and induces oxidative stress

**Figure 3:**
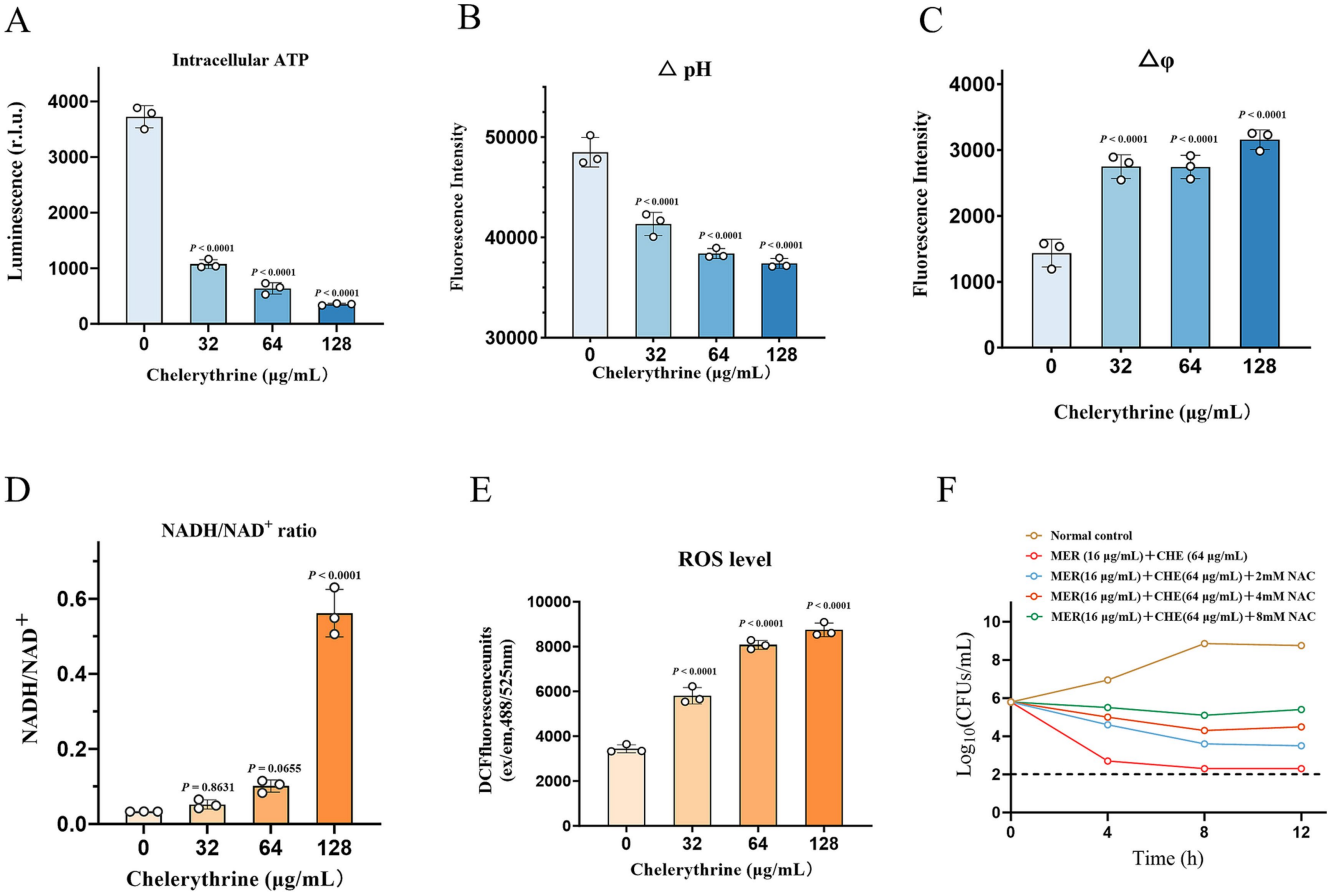
Chelerythrine disrupts bacterial energy metabolism and induces oxidative stress in *E. coli* B2. **(A–E)** *E. coli* B2 was treated with the indicated concentrations of chelerythrine (CHE), followed by measurement of intracellular ATP levels **(A)**, transmembrane pH gradient (ΔpH) **(B)**, membrane potential (Δψ) **(C)**, NADH/NAD + ratio **(D)**, and intracellular ROS levels **(E)**. ROS production was quantified using DCF fluorescence at Ex/Em 488/525 nm. **(F)** Effect of N-acetyl-L-cysteine (NAC)-mediated ROS scavenging on bacterial survival during combined meropenem (MER, 16 μg/mL) and CHE (64 μg/mL) treatment. The dashed line indicates the lower limit of detection. Data are presented as mean ± SD from three independent biological replicates. Statistical significance was determined by one-way ANOVA followed by Dunnett’s multiple-comparison test versus the untreated control; exact *p* values are shown in the figure.

To further understand the mechanism of chelerythrine as a meropenem adjuvant, its effect on bacterial energy metabolism was investigated. A dose–dependent reduction in intracellular ATP levels was observed upon chelerythrine treatment (Figure 3A). Given that ATP synthesis is propelled by the proton motive force (PMF), which comprises of membrane potential (Δψ) and pH gradient (ΔpH), Δψ and ΔpH levels were evaluated. Chelerythrine induced a concentration-dependent reduction in ΔpH (Figure 3B) and a compensatory elevation in Δψ (Figure 3C), suggesting disruption of the PMF.

The NADH/NAD^+^ ratio increased dramatically in a chelerythrine dose-dependent manner (Figure 3D). Consistent with the elevated NADH/NAD^+^ ratio, intracellular ROS levels increased in a concentration-dependent manner upon chelerythrine treatment (Figure 3E). The addition of the ROS scavenger NAC markedly diminished the bactericidal efficacy of the chelerythrine-meropenem combination (Figure 3F), indicating that chelerythrine-induced ROS contributed to the enhanced antibacterial activity. These findings demonstrated that chelerythrine potentiated the effect of meropenem by increasing oxidative damage and disrupting bacterial energy homeostasis.

### 3.4 Chelerythrine enhances meropenem efficacy *in vivo*

**Figure 4:**
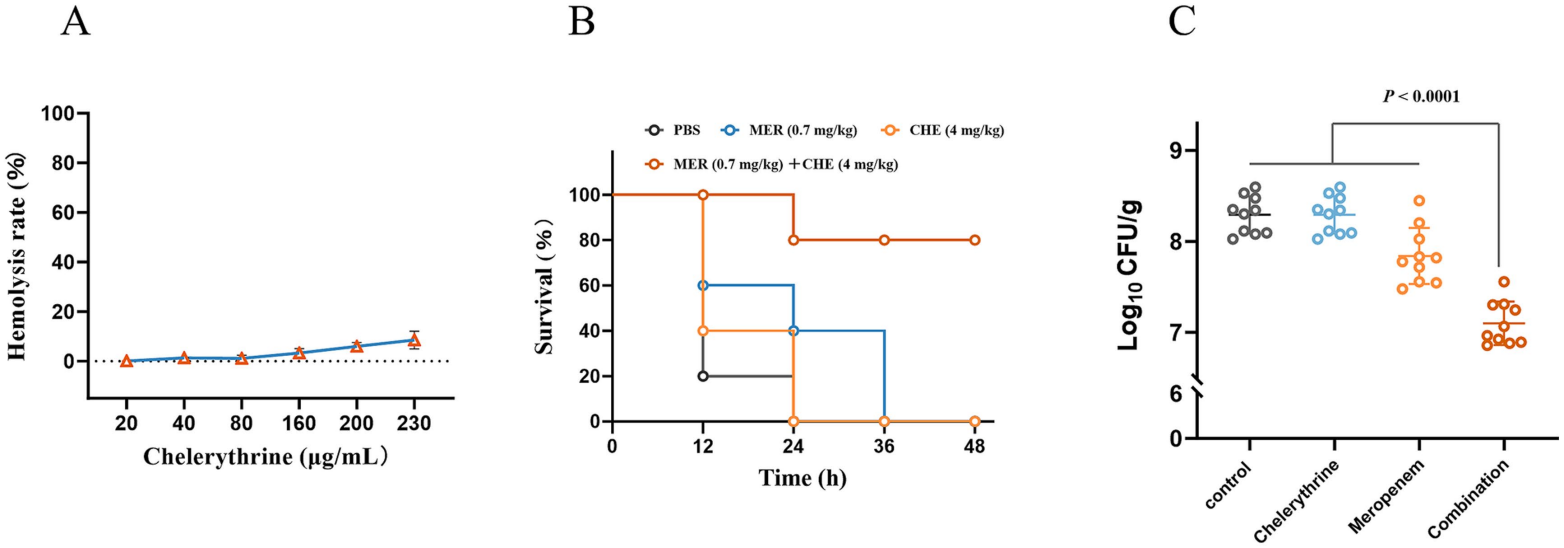
*In vivo* synergistic efficacy of chelerythrine and meropenem against *E. coli* B2 infection. **(A)** Hemolytic activity of chelerythrine against mammalian red blood cells. **(B)** Survival of Galleria mellonella larvae infected with *E. coli* B2 and treated with PBS, meropenem (MER), chelerythrine (CHE), or MER plus CHE. **(C)** Bacterial loads in thigh tissues from infected mice after treatment with vehicle, CHE, MER, or MER plus CHE. Data are presented as mean ± SD; statistical significance is indicated in the figure.

Considering the potent *in vitro* synergy between chelerythrine and meropenem, we subsequently evaluated their *in vivo* safety and efficacy. Hemolysis experiments indicated that chelerythrine caused negligible red blood cell lysis (<10%) at concentrations up to 230 μg/mL, implying satisfactory biocompatibility (Figure 4A).

The *in vivo* efficacy of the chelerythrine–meropenem combination was evaluated using a *Galleria mellonella* larval infection model. Larvae infected with *E. coli* B2 exhibited 100% mortality within 24 h in both the vehicle and chelerythrine monotherapy groups. All larvae in the meropenem monotherapy group died by 36 h. However, combination therapy yielded an 80% survival rate at 48 h (Figure 4B), demonstrating robust in vivo synergistic efficacy. This was further supported by a murine thigh infection model, in which combination therapy markedly diminished bacterial load (Figure 4C). These data collectively indicated that the combination of chelerythrine and meropenem exhibited excellent *in vivo* synergistic efficacy against NDM–producing Enterobacteriaceae.

## 4 Discussion

Carbapenems are last-resort drugs for multidrug-resistant Gram-negative infections (25), yet the global dissemination of blaNDM genes has fueled carbapenem resistance and intensified a One Health threat (26). NDM-producing Enterobacteriaceae have been detected in food-producing animals, companion animals, and the farm-to-food continuum, increasing the risk of cross-sector transmission. Antibiotic adjuvants, particularly NDM inhibitors, offer a cost-effective strategy to restore carbapenem activity and strengthen infection control in veterinary medicine (27, 28). This study found that chelerythrine, a natural benzophenanthridine alkaloid derived from *Chelidonium majus* and *Macleaya cordata*, exhibited significant synergistic antibacterial activity with meropenem against blaNDM–positive *E. coli* both *in vitro* and *in vivo.* Mechanistic investigations revealed that chelerythrine potentiated meropenem efficacy by suppressing NDM-mediated hydrolysis and increasing oxidative damage. These results provide a promising adjuvant for meropenem in combating NDM-mediated resistance.

Chelerythrine enhanced the bactericidal effect of meropenem by inhibiting NDM, a among mechanism of carbapenem adjuvants. Venkata et al. (37) summarized NDM-1 inhibitors reported before 2021, including chelating agents, thiols, thioesters, carboxylic acids, bisthiazolidines, cyclic boronates, phosphonates, and heterocyclic derivatives, among others. However, chelerythrine, identified in this study as a benzophenanthridine alkaloid, is structurally distinct from previously reported NDM-1 inhibitors, suggesting that benzophenanthridine alkaloids may serve as a novel scaffold for NDM-1 inhibitor development.

The computational analyses performed in this study suggest that chelerythrine can stably occupy the catalytic pocket of NDM-1 and preferentially bind to the dinuclear zinc-containing active-site region during the MD simulation. Given that NDM-1 catalytic activity depends on the integrity of the zinc-centered catalytic microenvironment and the precise spatial arrangement of surrounding residues, stable binding of chelerythrine in this region may interfere with substrate accommodation and catalytic turnover. Docking analysis further indicated that chelerythrine preferentially adopts a binding mode adjacent to the metal center, with its binding stabilized by polar interactions, particularly with His250 and Asn220, as well as hydrophobic contacts involving residues such as Val73 and Trp93. These results support a model in which chelerythrine inhibits NDM primarily by occupying the catalytic pocket and interacting with residues surrounding the dinuclear zinc center, thereby potentially perturbing the local geometry and physicochemical environment required for efficient hydrolysis.

Molecular dynamics simulations further supported this interpretation, showing that the chelerythrine–NDM-1 complex remained globally stable without evidence of large-scale structural destabilization. These findings suggest that chelerythrine inhibits NDM primarily by impairing catalytic activity rather than by altering the overall protein fold, thereby preserving the activity of meropenem against penicillin-binding proteins (PBPs) and consequently inhibiting peptidoglycan polymerization and cross-linking.

Moreover, chelerythrine combined with meropenem also exhibited synergistic activity against bacteria producing other Zn^2+^-dependent metallo-*β*-lactamases, including IMP-4, IMP-10, and VIM-1, with particularly strong synergy observed against the VIM-1-producing strain (FICI = 0.187). In contrast, the combination showed only weak or no synergistic activity against strains producing non-Zn^2+^-dependent carbapenemases, including *Klebsiella pneumoniae* carbapenemase-2 (KPC-2) and the OXA-48-like class D serine carbapenemase OXA-181, especially against the OXA-181-producing strain (FICI = 1). These results support the notion that chelerythrine potentiates meropenem, at least in part, by interfering with the Zn^2+^-dependent catalytic function of metallo-*β*-lactamases. This mode of action is consistent with previous reports on metallo-β-lactamase inhibitors. For example, King et al. (29) reported in Nature that aspergillomarasmine A (AMA) inhibits NDM-1, VIM-2, and IMP-7 by removing metal ions from the active sites of these enzymes. Subsequent studies further demonstrated that AMA acts as a selective Zn^2+^ scavenger and promotes the dissociation of one Zn^2+^ cofactor from NDM-1, thereby indirectly inactivating the enzyme. Similarly, dithiocarbamate-containing compounds possess strong metal-coordinating capacity and can inhibit β-lactam hydrolysis by forming coordination interactions with the catalytic Zn^2+^ ions in the active site of metallo-β-lactamases (30).

**Figure 5:**
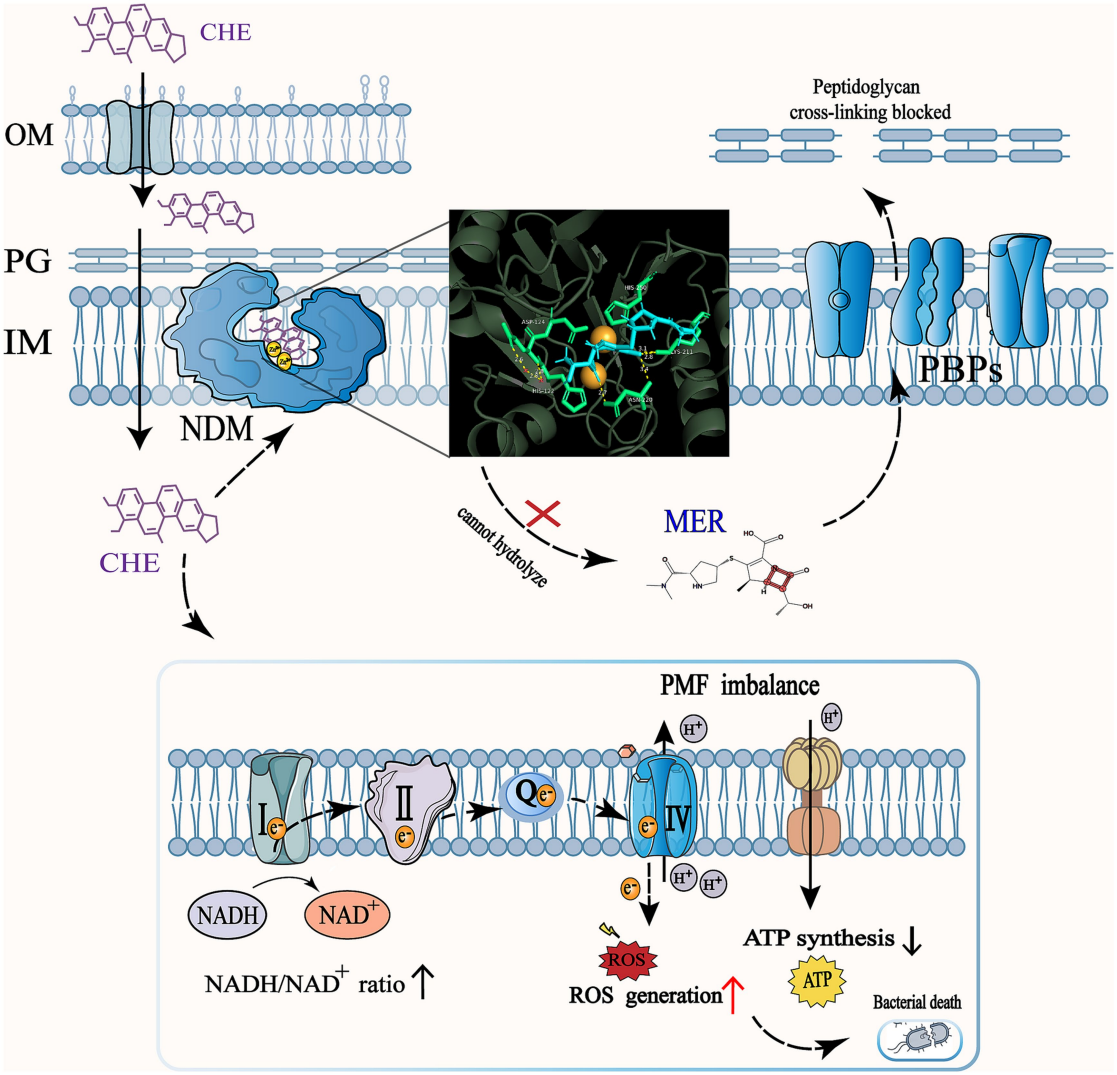
Mechanism diagram of the synergistic effect between meropenem and chelerythrine.

Notably, chelerythrine displayed more pronounced inhibitory activity against NDM-producing bacteria than against strains carrying IMP-type Zn^2+^-dependent metallo-*β*-lactamases, including IMP-4 and IMP-10, with FICI values of ≤0.375 for NDM-producing strains. Similar selectivity has also been reported for other NDM potentiators. For instance, isoliquiritin restored meropenem activity against NDM-1-positive *E. coli* and *K. pneumoniae* with FICI values below 0.5, whereas it showed no obvious inhibitory activity against VIM-1 or NDM-5 (31). Li et al. (32) reported that PHT427 may interact with the Zn1 and Zn2 sites of NDM-1 while forming stable interactions with active-site residues such as Asn220 and Gln123. The inhibitory effect of PHT427 was partially reversed by Zn^2+^ supplementation, suggesting that its activity may involve both perturbation of the Zn^2+^-dependent catalytic center and NDM-1-specific active-site residue interactions. Together, these findings further support a structurally specific interaction mechanism between chelerythrine and NDM enzymes (Figure 5). In addition, the pronounced synergistic antibacterial activity observed between resveratrol and meropenem against VIM-1-producing bacteria suggests that the potentiation of carbapenems against distinct metallo-*β*-lactamase subtypes may involve enzyme-specific mechanisms that warrant further investigation.

Beyond NDM inhibition, chelerythrine potentiates meropenem-mediated killing by disrupting bacterial respiratory and bioenergetic homeostasis. The elevated intracellular NADH/NAD^+^ ratio is indicative of impaired NADH oxidation and a pronounced redox imbalance. This perturbation likely compromises proton translocation across the membrane, leading to dissipation of ΔpH and an abnormal increase in Δψ, a profile more consistent with proton motive force (PMF) disequilibrium than with effective membrane energization. Consistent with this bioenergetic defect, ATP production was significantly diminished. Impaired electron transport may enhance electron leakage to oxygen, resulting in excessive ROS generation. ROS appeared to be a key contributor to synergistic killing, as evidenced by the reduced efficacy of the chelerythrine–meropenem combination upon addition of the ROS scavenger NAC. A role for ROS in bactericidal activity has been supported by several studies; for example, aminoglycosides and quinolones have been reported to partially exert bactericidal effects through ROS-mediated damage (33). The antibacterial activities of isobavachalcone and melatonin have been linked to intracellular ROS accumulation. Consistent with this mechanism, chelerythrine has also been reported to potentiate colistin against mcr-1–positive Enterobacteriaceae (19, 34). These findings highlight the potential of chelerythrine as an antibiotic adjuvant scaffold, particularly for difficult-to-treat Gram–negative infections.

The therapeutic potential of the chelerythrine–meropenem combination was validated in both *Galleria mellonella* and murine infection models. Chelerythrine not only potentiated the antibacterial activity of meropenem but also delayed the emergence of meropenem resistance, highlighting its potential to extend the therapeutic utility of meropenem against NDM-producing pathogens. Beyond efficacy, safety is a key consideration for further translation. In this study, chelerythrine showed negligible hemolytic activity at effective concentrations, supporting its preliminary biocompatibility.

This observation is also consistent with previous mammalian studies showing that chelerythrine can be tolerated in short-term *in vivo* settings under specific experimental conditions. For example, oral administration of chelerythrine at 1–10 mg/kg in a lipopolysaccharide-induced endotoxic shock model, and at 5–10 mg/kg in an acute lung injury model, protected mice by reducing inflammatory cytokine production, neutrophil infiltration, oxidative stress, and NF-κB activation, without reporting overt acute toxicity under these conditions (35, 36). Although the present findings provide preliminary evidence of biocompatibility, comprehensive safety evaluation remains insufficient. Future studies should therefore further define the therapeutic window, tissue distribution, repeated-dose toxicity, and PK/PD relationship of chelerythrine, both alone and in combination with meropenem, in target animal species.

In conclusion, this study demonstrated that chelerythrine, a natural benzophenanthridine alkaloid, potentiated the antibacterial activity of meropenem against NDM–producing Enterobacteriaceae both *in vitro* and *in vivo*. Chelerythrine not only inhibited NDM enzymatic activity by occupying the catalytic pocket and interacting with residues surrounding the dinuclear zinc center, but also disrupted bacterial energy metabolism and induced oxidative stress. This study provides a promising therapeutic strategy against carbapenem-resistant Enterobacteriaceae in veterinary settings and underscores the potential of natural compounds as antibiotic adjuvants.

## Data Availability

The data that support the findings of this study are available from the corresponding author upon reasonable request.

## References

[1] Perez F El Chakhtoura NG Papp-Wallace KM Wilson BM Bonomo RA. Treatment options for infections caused by carbapenem-resistant Enterobacteriaceae: can we apply “precision medicine” to antimicrobial chemotherapy? Expert Opin Pharmacother. (2016) 17:761–81. doi: 10.1517/14656566.2016.1145658 26799840 PMC4970584

[2] Gupta N Limbago BM Patel JB Kallen AJ. Carbapenem-resistant Enterobacteriaceae: epidemiology and prevention. Clin Infect Dis. (2011) 53:60–7. doi: 10.1093/cid/cir202, 21653305

[3] Potter RF D’Souza AW Dantas J. The rapid spread of carbapenem-resistant Enterobacteriaceae. Drug Resist Updat. (2016) 29:30–46. doi: 10.1016/j.drup.2016.09.002, 27912842 PMC5140036

[4] Shen Y Hu F Wang Y Yin D Yang L Chen Y . Transmission of carbapenem resistance between human and animal NDM-positive Escherichia coli strains. Engineering. (2022) 15:24–33. doi: 10.1016/j.eng.2021.07.030

[5] Aurilio C Sansone P Barbarisi M Pota V Giaccari LG Coppolino F . Mechanisms of action of carbapenem resistance. Antibiotics. (2022) 11:421. doi: 10.3390/antibiotics11030421, 35326884 PMC8944602

[6] Logan LK Weinstein RA. The epidemiology of carbapenem-resistant Enterobacteriaceae: the impact and evolution of a global menace. J Infect Dis. (2017) 215:S28–36. doi: 10.1093/infdis/jiw282, 28375512 PMC5853342

[7] Papagiannitsis CC Pollini S De Luca F Rossolini GM Docquier J-D Hrabák J. Biochemical characterization of VIM-39, a VIM-1-like metallo-β-lactamase variant from a multidrug-resistant Klebsiella pneumoniae isolate from Greece. Antimicrob Agents Chemother. (2015) 59:7811–4. doi: 10.1128/aac.01935-15, 26369975 PMC4649142

[8] Poirel L Nordmann P. Carbapenem resistance in Acinetobacter baumannii: mechanisms and epidemiology. Clin Microbiol Infect. (2006) 12:826–36. doi: 10.1111/j.1469-0691.2006.01456.x 16882287

[9] Lewis K. The science of antibiotic discovery. Cell. (2020) 181:29–45. doi: 10.1016/j.cell.2020.02.056, 32197064

[10] Årdal C Balasegaram M Laxminarayan R McAdams D Outterson K Rex JH . Antibiotic development—economic, regulatory and societal challenges. Nat Rev Microbiol. (2020) 18:267–74. doi: 10.1038/s41579-019-0293-3 31745330

[11] Gülen D Şafak B Erdal B Günaydın B. Curcumin-meropenem synergy in carbapenem resistant Klebsiella pneumoniae curcumin-meropenem synergy. Iran J Microbiol. (2021) 13:345. doi: 10.18502/ijm.v13i3.6397 34540173 PMC8416589

[12] Wu S Wei Y Wang Y Zhang Z Liu D Qin S . Liposomal antibiotic booster potentiates carbapenems for combating NDMs-producing Escherichia coli. Adv Sci. (2024) 11:2304397. doi: 10.1002/advs.202304397 PMC10787095 37933983

[13] Guo Y Yang Y Xu X Li L Zhou Y Jia G . Metallo-β-lactamases inhibitor fisetin attenuates meropenem resistance in NDM-1-producing Escherichia coli. Eur J Med Chem. (2022) 231:114108. doi: 10.1016/j.ejmech.2022.114108 35101651

[14] Zhou Y Lv X Chen M Guo Y Ding R Liu B . Characterization of corosolic acid as a KPC-2 inhibitor that increases the susceptibility of KPC-2-positive bacteria to carbapenems. Front Pharmacol. (2020) 11:1047. doi: 10.3389/fphar.2020.01047 32733256 PMC7363806

[15] Niu XF Zhou P Li WF Xu HB. Effects of chelerythrine, a specific inhibitor of cyclooxygenase-2, on acute inflammation in mice. Fitoterapia. (2011) 82:620–5. doi: 10.1016/j.fitote.2011.01.020, 21291962

[16] Colombo ML Bosisio EJP. Pharmacological activities of chelidonium majus L. (papaveraceae). Pharmacol Res. (1996) 33:127–34. doi: 10.1006/phrs.1996.0019 8870028

[17] Chmura SJ Dolan ME Cha A Mauceri HJ Kufe DW Weichselbaum RR. In vitro and in vivo activity of protein kinase C inhibitor chelerythrine chloride induces tumor cell toxicity and growth delay in vivo. Clin Cancer Res. (2000) 6:737–42. 10690561

[18] He N Wang P Wang P Ma C Kang W. Antibacterial mechanism of chelerythrine isolated from root of Toddalia asiatica (Linn) lam. BMC Complement Altern Med. (2018) 18:261. doi: 10.1186/s12906-018-2317-3 30257662 PMC6158911

[19] Song H Wang X Zhang M Zou Z Yang S Yi T . Dual effects of feed-additive-derived chelerythrine in combating mobile colistin resistance. Engineering. (2024) 32:163–73. doi: 10.1016/j.eng.2023.06.012

[20] Zhang H Jiang L Zhao Y He M Wang Z Liu YJ. A novel ABC family protein participates in the regulation of fitness cost caused by tet (X4)-bearing plasmids in Escherichia coli. Fundam Res. (2024) 6:895–905. doi: 10.1016/j.fmre.2024.03.020 41971834 PMC13069663

[21] Feng H Liu X Wang S Fleming J Wang D-C Liu W. The mechanism of NDM-1-catalyzed carbapenem hydrolysis is distinct from that of penicillin or cephalosporin hydrolysis. Nat Commun. (2017) 8:2242. doi: 10.1038/s41467-017-02339-w, 29269938 PMC5740130

[22] Wang R Lai T-P Gao P Zhang H Ho P-L Woo PC-Y . Bismuth antimicrobial drugs serve as broad-spectrum metallo-β-lactamase inhibitors. Nat Commun. (2018) 9:439. doi: 10.1038/s41467-018-02828-6, 29382822 PMC5789847

[23] Ma B Fang C Lu L Wang M Xue X Zhou Y . The antimicrobial peptide thanatin disrupts the bacterial outer membrane and inactivates the NDM-1 metallo-β-lactamase. Nat Commun. (2019) 10:3517. doi: 10.1038/s41467-019-11503-3, 31388008 PMC6684654

[24] González LJ Bahr G Nakashige TG Nolan EM Bonomo RA Vila AJ. Membrane anchoring stabilizes and favors secretion of New Delhi metallo-β-lactamase. Nat Chem Biol. (2016) 12:516–22. doi: 10.1038/nchembio.2083, 27182662 PMC4912412

[25] Kim HR Eom YBJJ. Auranofin promotes antibacterial effect of doripenem against carbapenem-resistant Acinetobacter baumannii. J Appl Microbiol. (2022) 133:1422–33. doi: 10.1111/jam.15644, 35633297

[26] Li W Dong K Ren J Qu X. A β-lactamase-imprinted responsive hydrogel for the treatment of antibiotic-resistant bacteria. Angew Chem. (2016) 128:8181–5. doi: 10.1002/ange.201600205, 27159893

[27] Ejim L Farha MA Falconer SB Wildenhain J Coombes BK Tyers M . Combinations of antibiotics and nonantibiotic drugs enhance antimicrobial efficacy. Nat Chem Biol. (2011) 7:348–50. doi: 10.1038/nchembio.559, 21516114

[28] Drawz SM Bonomo RA. Three decades of β-lactamase inhibitors. Clin Microbiol Rev. (2010) 23:160–201. doi: 10.1128/cmr.00037-09, 20065329 PMC2806661

[29] King AM Reid-Yu SA Wang W King DT De Pascale G Strynadka NC . Aspergillomarasmine a overcomes metallo-β-lactamase antibiotic resistance. Nature. (2014) 510:503–6. doi: 10.1038/nature13445, 24965651 PMC4981499

[30] Wang M-M Chu W-C Yang Y Yang Q-Q Qin S-S Zhang E. Dithiocarbamates: efficient metallo-β-lactamase inhibitors with good antibacterial activity when combined with meropenem. Bioorg Med Chem Lett. (2018) 28:3436–40. doi: 10.1016/j.bmcl.2018.09.028, 30262427

[31] Wang Y Sun X Kong F Xia L Deng X Wang D . Specific NDM-1 inhibitor of isoliquiritin enhances the activity of meropenem against NDM-1-positive Enterobacteriaceae in vitro. Int J Environ Res Public Health. (2020) 17:2162. doi: 10.3390/ijerph17062162, 32213926 PMC7143545

[32] Li X Wang Q Zheng J Guan Y Liu C Han J . PHT427 as an effective New Delhi metallo-β-lactamase-1 (NDM-1) inhibitor restored the susceptibility of meropenem against Enterobacteriaceae producing NDM-1. Front Microbiol. (2023) 14:1168052. doi: 10.3389/fmicb.2023.1168052, 37138606 PMC10150926

[33] Van Acker H Coenye T Tim J. The role of reactive oxygen species in antibiotic-mediated killing of bacteria. Trends Microbiol. (2017) 25:456–66. doi: 10.1016/j.tim.2016.12.008 28089288

[34] Wei X Guo J Geng X Xue B Huang S Yuan Z. The combination of membrane disruption and FtsZ targeting by a chemotherapeutic hydrogel synergistically combats pathogens infections. Adv Healthc Mater. (2024) 13:2304600. doi: 10.1002/adhm.202304600 38491859

[35] Niu X Mu Q Li W Huang H Yao H Li H. Protective effects of chelerythrine against lipopolysaccharide-induced endotoxic shock in mice. Inflammation. (2014) 37:1968–75. doi: 10.1007/s10753-014-9929-7 24928629

[36] Fan L Fan Y Liu L Tao W Shan X Dong Y . Chelerythrine attenuates the inflammation of lipopolysaccharide-induced acute lung inflammation through NF-κB signaling pathway mediated by Nrf2. Front Pharmacol. (2018) 9:1047. doi: 10.3389/fphar.2018.01047, 30319404 PMC6169195

[37] Venkata KCN Ellebrecht M Tripathi SK. Efforts towards the inhibitor design for New Delhi metallo-beta-lactamase (NDM-1). Eur. J. Med. Chem. (2021) 225:113747. 34391033 10.1016/j.ejmech.2021.113747

