# Supplementary material for: Chelerythrine potentiates meropenem activity against NDM-producing carbapenem-resistant Enterobacteriaceae
Source: Front Vet Sci. 2026 Jul 8;13:1878928. doi: 10.3389/fvets.2026.1878928 (PMC13388087; doi:10.3389/fvets.2026.1878928)
Supplement: Supplementary file 1 [file Supplementary_file_1.pdf]

Supplementary Material

Bacterial strain (resistance gene)	meropenem MIC (μg/mL)
E. coli M593(*bla*$_{NDM-1}$)	16
E. coli B2(*bla*$_{NDM-5}$)	32
E. coli HH194(*bla*$_{NDM-9}$)	32
*E.coli*DH5α(*bla*$_{VIM-1}$)	4
E. coli DH5α (*bla*$_{KPC-2}$)	16
E. coli DH5α (*bla*$_{IMP-4}$)	6
*P. aeruginosa*PA22-40(*bla*$_{IMP-10}$)	128
*K. oxytoca*BT6-1 (*bla*$_{OXA-181}$)	64
K.pneumoniaeLR1-1-1(blaNDM-5)	32
K.pneumoniaeLR11-2(blaNDM-1)	32
E. coli DH5α	0.125
E. coli K582	0.125
ATCC 25922	0.125

Table S1. Meropenem susceptibility profiles of bacterial strains carrying different *β*-lactamase resistance genes. The resistance gene carried by each strain is indicated in parentheses. Meropenem MIC values are expressed as μg/mL. MIC, minimum inhibitory concentration.

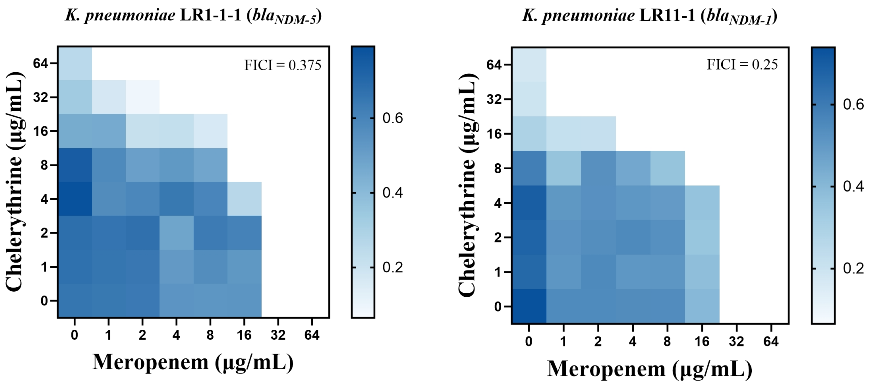

Figure S1. Synergistic activity of chelerythrine and meropenem against NDM-producing Klebsiella pneumoniae strains. Representative checkerboard assays showing the synergistic antibacterial activity of chelerythrine (CHE) combined with meropenem (MER) against NDM-producing *K. pneumoniae* strains. The fractional inhibitory concentration index (FICI) values were 0.375 and 0.25, respectively, indicating synergistic interactions between CHE and MER.

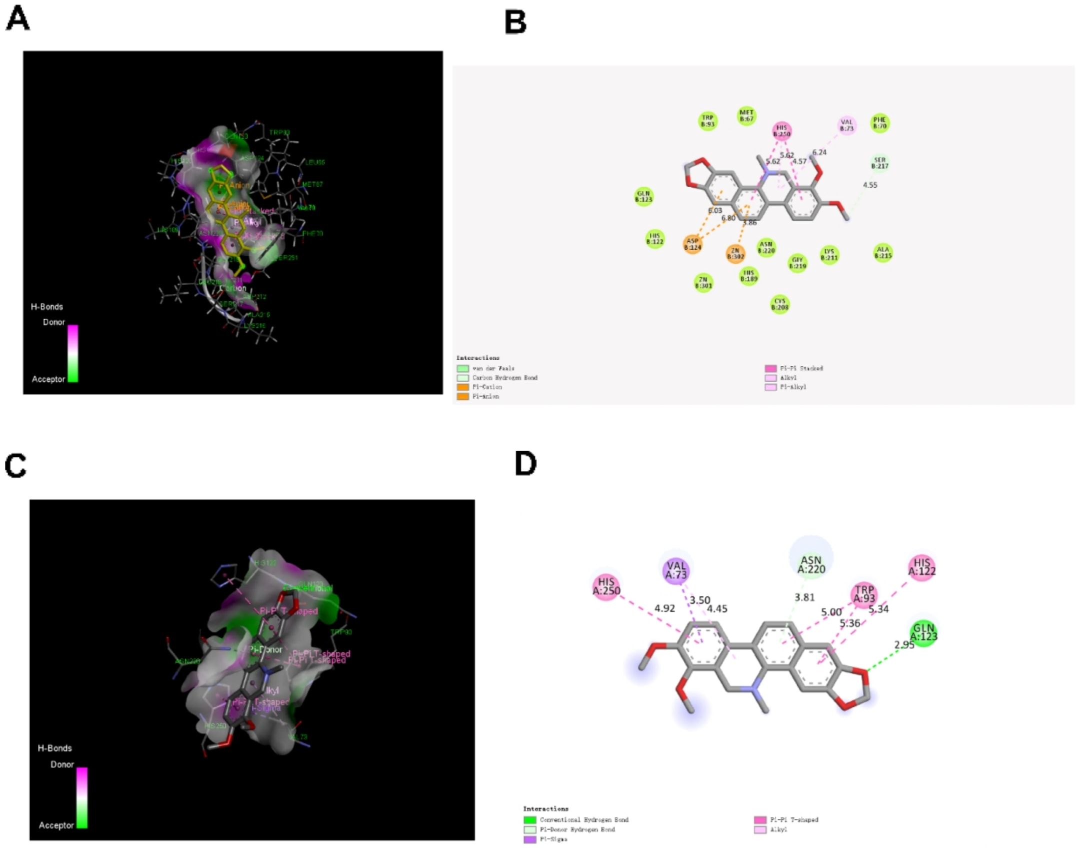

Figure S2. Molecular docking analysis of chelerythrine with NDM-5 and NDM-9. (A, B) Predicted binding mode of chelerythrine (CHE) in the substrate-accessible catalytic pocket of NDM-5, adjacent to the dinuclear Zn^{2+} center, with a binding energy of −6.744 kcal/mol. CHE forms close contacts with key active-site residues, including His122, Asp124, His189, Lys211, Asn220, and His250. (C, D) Predicted binding mode of CHE in the catalytic pocket of NDM-9, with a binding energy of −7.376 kcal/mol. CHE is positioned near the dinuclear Zn^{2+} center and interacts with residues including Met67, Val73, Trp93, His122, Gln123, Asp124, Asn220, and His250. These docking results suggest that CHE may inhibit NDM activity by occupying the substrate-binding pocket and perturbing the catalytic microenvironment.
